# New insights into imaging of pulmonary metastases from extra-thoracic neoplasms

**DOI:** 10.1007/s11547-025-02008-9

**Published:** 2025-04-01

**Authors:** Giuseppe Cicchetti, Riccardo Marano, Cecilia Strappa, Silvia Amodeo, Alessandro Grimaldi, Ludovica Iaccarino, Francesco Scrocca, Leonardo Nardini, Annachiara Ceccherini, Annemilia Del Ciello, Alessandra Farchione, Luigi Natale, Anna Rita Larici

**Affiliations:** 1 Advanced Radiology Center, Department of Diagnostic Imaging and Radiation Oncology, Fondazione Policlinico Universitario A. Gemelli IRCCS, L.go A. Gemelli, 8, 00168 Rome, Italy; 2 Section of Radiology, Department of Radiological and Hematological Sciences, Università Cattolica del Sacro Cuore, Rome, Italy

**Keywords:** Pulmonary nodules, Metastasis, Cancer, Differential diagnoses, Multimodal imaging, Management

## Abstract

The lung is one of the most common sites of metastases from extra-thoracic neoplasms. Lung metastases can show heterogeneous imaging appearance, thus mimicking a wide range of lung diseases, from benign lesions to primary lung cancer. The proper interpretation of pulmonary findings is crucial for prognostic assessment and treatment planning, even to avoid unnecessary procedures and patient anxiety. For this purpose, computed tomography (CT) is one of the most used imaging modalities. In the last decades, cancer patients’ population has steadily increased and, due to the widespread application of CT for staging and surveillance, the detection of pulmonary nodules has raised, making their characterization and management an urgent and mostly unsolved problem for both radiologists and clinicians. This review will highlight the pathways of dissemination of extra-thoracic tumours to the lungs and the heterogeneous CT imaging appearance of pulmonary metastases, providing useful clues to properly address the diagnosis. Furthermore, we will deal with the promising applications of radiomics in this field. Finally, a focus on the hot-topic of pulmonary nodule management in patients with extra-thoracic neoplasms (ETNs) will be discussed.

## Introduction

Pulmonary metastases from extra-thoracic neoplasms (ETNs) are the most frequent malignant lung tumours [1]. The early recognition of pulmonary metastases and their differentiation from other entities is essential to properly address the diagnosis and further patient’s management, as well as limiting unnecessary diagnostic procedures and treatment. Thus, radiologists need to be aware of the possible imaging appearance of pulmonary metastases and non-neoplastic diseases that may mimic lung metastatic spread of ETN, especially on chest computed tomography (CT), which currently represents the imaging modality of choice for both identification and characterization of pulmonary lesions.

Over the last decades, the improvement in early diagnosis and the availability of effective cancer treatment strategies have led to a better life expectancy for oncologic patients. Additionally, due to the widespread use of CT imaging in oncology for staging and follow-up purposes, detection of pulmonary nodules in cancer patients has dramatically increased [2]. In this population, nodules may represent not only metastases, but also benign processes or even lung cancer, which is the most frequent second primary tumour [3–5]. Imaging can provide useful clues to assess the probability of pulmonary nodule malignancy. However, the lack of accepted guidelines, which reflects the fragmentary and non-univocal results in the literature available so far, complicates nodule management in this population and makes radiologists’ task as hard as pivotal.

The aim of this review is to outline the pathways of dissemination of ETNs to the lungs, depicting the range of radiological appearances of pulmonary metastases and their main differential diagnoses, and to provide useful clues to improve diagnostic accuracy in this complex issue. In the final sections we will deal with the promising applications of radiomics in pulmonary nodule characterization as well as we will focus on the challenging topic of pulmonary nodules management in patients with ETNs.

### Epidemiology

The lung is one of the most common sites of distant metastases from ETNs [1]. According to autoptic series performed on patients who died from malignancy, lung metastases have been demonstrated in up to 54% of cases [6–8]. In a fairly recent study on 3827 autopsies, the lung was the second most common distant metastatic site after the liver, with a frequency of 10.7% and 11.1%, respectively [9]. In addition, metastases from ETNs are the most common malignant pulmonary lesions [1]. Among ETNs, colorectal, kidney, pancreatic and breast cancers are the most common causes of pulmonary metastases [10]. In the literature, the incidence of pulmonary metastases varies due to different definitions applied in clinical studies; nonetheless, combined data from multiple autopsy series describe the cumulative incidence of pulmonary metastases, expressed as the percentage of patients with a specific tumour histology [1, 11]. Sarcoma, renal cell carcinoma, melanoma and adrenocortical cancer more likely show metastatic spread to the lungs (55–95% of metastatic cases), followed by neoplasms of testes (40–50%), liver (37–70%), pancreas (40%), head and neck (12–41%), ovaries (28%), prostate (25%), thyroid (10–25%), colon-rectum (14%), and breast (5%) [1, 11, 12].

### Lung dissemination patterns

The most common pattern of dissemination of ETNs to the lungs is the *hematogenous spread*, either arterial or venous, followed by lymphatic and endobronchial routes [1, 13]. This is due to some distinctive features of the pulmonary vascular system. Indeed, lungs receive the entire cardiac output every minute and have the densest capillary bed in the whole body [1]; in addiction, pulmonary arterial system represents the primary capillary filter for the drainage of most organs [14]. However, when dealing with pulmonary metastatic spread, it must be taken into consideration that a combination of different pathways of dissemination is possible [13].

**Fig. 1:**
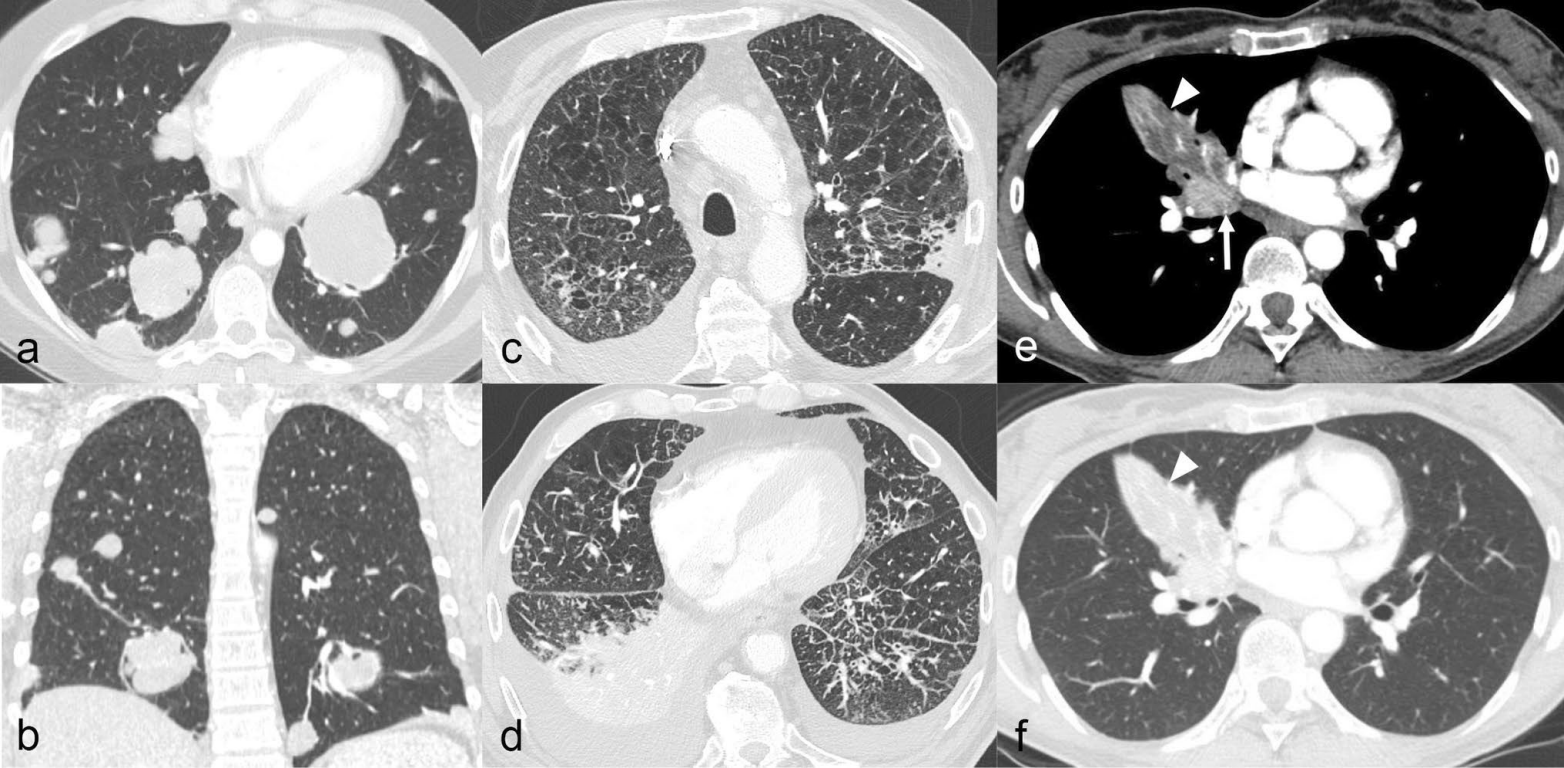
Typical CT appearance and distribution of hematogenous pulmonary metastases, lymphangitic carcinomatosis and endobronchial metastasis. **a**, **b** Lung metastases in a 32 y.o. woman affected by Ewing sarcoma. Axial (**a**) and coronal (**b**) computed tomography (CT) images show multiple solid nodular and rounded lesions of variable size, preferentially located in the basal regions of the lungs. **c**, **d** Lymphangitic carcinomatosis in a 74 y.o. man affected by advanced stage colon cancer. Axial CT images show bilateral smooth thickening of the interlobular septa and peribronchovascular interstitium, predominant in the lung bases, associated with bilateral pleural effusion and atelectasis of the right lower lobe. Note the absence of architectural distortion of the secondary pulmonary lobules. **e**, **f** Endobronchial metastasis in a 57 y.o. patient with melanoma (**e**, **f**). Contrast-enhanced CT images show a well-defined, homogeneous and strongly enhancing solid nodule (white arrows in **e**), that obstructs the right middle lobe bronchus, determining distal lobar atelectasis (arrowheads in **e**, **f**)

Hematogenous spread derives from the detachment of cells from the primary tumour, with endovascular invasion and seeding into the pulmonary capillary bed [1, 6]. Accordingly, hematogenous metastases, which usually appear as solid nodules of variable number and size or even masses, are more conspicuous in the lower and peripheral lung regions, since these represent the more vascularized areas [6] (Fig. 1a, b). Hematogenous spread is the preferential pathway of pulmonary dissemination of malignant tumours of the head and neck, thyroid, adrenals, kidneys, and testes, because these organs own a direct venous drainage into the lung [15]. Furthermore, melanoma and osteosarcoma demonstrate this route of dissemination to the lungs [15].

*Lymphangitic carcinomatosis* refers to tumour growth in the pulmonary lymphatic channels, and more commonly represents a hematogenous dissemination with a subsequent extension to the lymphatics [16, 17]. First, malignant cells spread to pulmonary arterioles and capillaries, subsequently infiltrating and spreading within the lymphatics located along the axial (peribronchovascular and centrilobular) and peripheral (interlobular and subpleural) interstitial compartments. On thin-section CT, lymphangitic carcinomatosis shows the typical smooth and/or nodular thickening of the axial and peripheral interstitium, without architectural distortion of the secondary pulmonary lobules (Fig. 1c, d). Among the others, gastric, breast, pancreas, rectum, and prostate cancers most commonly demonstrate a lymphatic dissemination pathway [18].

*Endobronchial metastases* are more often the result of lymphatic or hematogenous spread to the bronchial walls rather than the direct tracheobronchial extension from parenchymal or nodal metastases [13, 19]. They can be directly visualized on CT scans as nodules located into the bronchial lumen, eventually causing atelectasis of the downstream lung parenchyma (sublobar, lobar, or involving the entire lung) [13, 19] (Fig. 1e, f). Endobronchial metastatic lesions most commonly originate from colorectal and renal cancer, but they can also be found in lymphomas [6].

## Imaging CT features of pulmonary metastases and differential diagnoses

Depending on the primary tumour histology and its preferred route of dissemination, lung metastases may present a wide range of imaging appearances on CT. Based on these imaging features, a diverse array of pulmonary diseases—either malignant or benign—may serve as differential diagnoses with pulmonary metastasis. In this section, the various CT appearance of pulmonary metastases will be illustrated, each accompanied by its main differential diagnoses.

### Nodules

The most common radiological appearance of lung metastases consists of multiple solid, well-circumscribed, rounded- or oval-shaped nodules of variable number and size. According to autopsy specimens, nodules multiplicity is detected in 75% of cases, with a predominant basal distribution, and 82–92% of metastases are located at the lung periphery [16, 20]. The *feeding vessel sign*, defined as the presence of a pulmonary vascular branch that runs towards the nodule, is frequently observed [21]. In this context, it is important to consider that a multinodular pattern may also be a manifestation of different benign disorders, such as organizing pneumonia (OP) and sarcoidosis, or of a pre-invasive neuroendocrine cell proliferation, called diffuse idiopathic pulmonary neuroendocrine cell hyperplasia (DIPNECH).

**Fig. 2:**
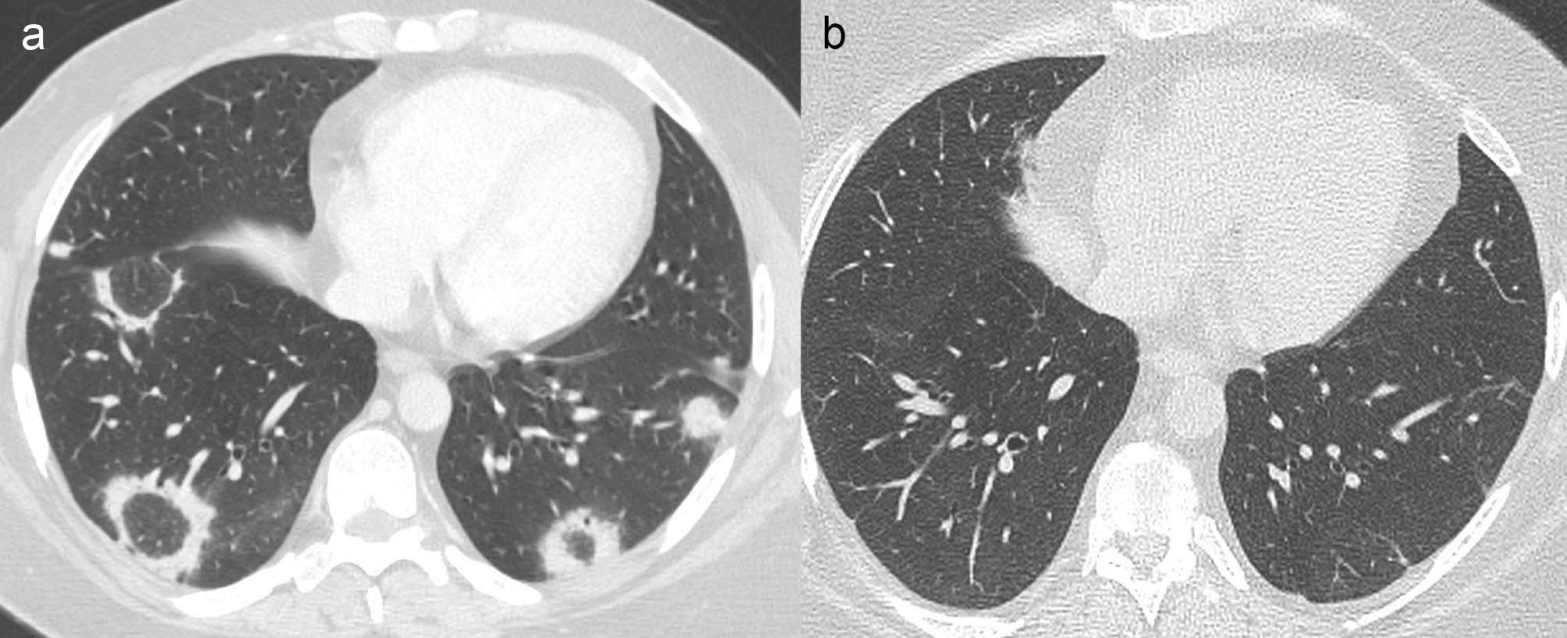
Drug-induced organizing pneumonia in an oncologic patient, with complete resolution after treatment. Drug-induced organizing pneumonia in a 29 y.o. woman with melanoma treated with nivolumab. Axial computed tomography (CT) image **a** shows bilateral perilobular consolidations with peripheral distribution in the lung bases. Axial CT image **b** performed after corticosteroid treatment shows almost complete resolution of the abnormalities, with some residual hazy ground glass opacities and subtle bands in the lower lobes

*OP* represents an important differential diagnosis, not only because its radiological appearance may mimic pulmonary metastases, but also because oncologic patients may develop OP during and after treatment with chemotherapy, radiation therapy, bone marrow and solid organ transplant [22]. Particularly, after radiation therapy in patients affected by breast cancer*,* OP may occur (2.5% of cases) with infiltrates located outside the irradiated volume, usually within 9 and 16 months after treatment completion [23]. OP may be detected even in patients treated with molecular targeting agents and immune checkpoint inhibitors, as the result of lung toxicity induced by immune-related adverse effects, including autoimmune response [24]. Although OP commonly presents with patchy unilateral or bilateral consolidation or ground-glass opacities, nodular opacities measuring 1–10 mm can be detected in 15–50% of patients [25, 26]. Nodules usually demonstrate a bilateral, scattered or peribronchovascular distribution, and in most cases are solid or part-solid [27–29]. Unlike pulmonary metastases, OP nodules may show ill-defined margins and may be associated with an open bronchus sign; these features, along with the propensity of nodules to fluctuate and migrate over time, sometimes resolving spontaneously, can strongly indicate a diagnosis of OP [27] (Fig. 2).

**Fig. 3:**
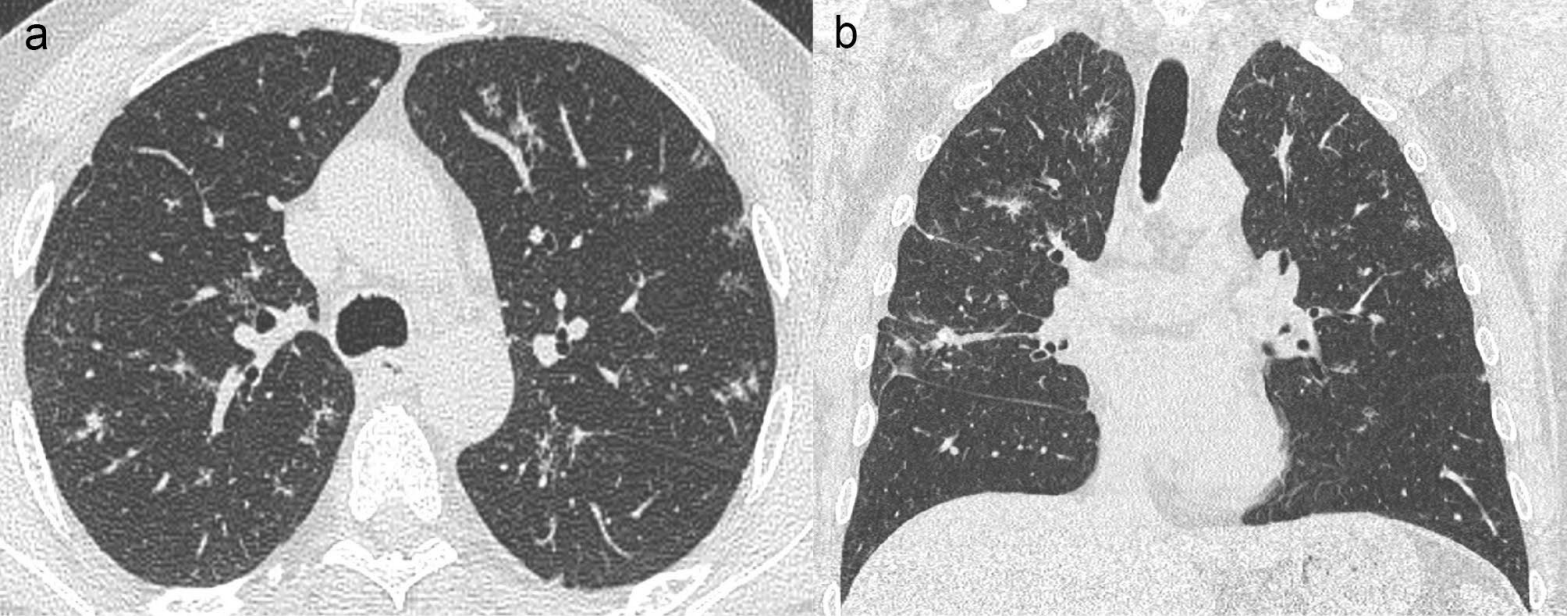
Pulmonary macronodular sarcoidosis in a patient affected by an extra-thoracic neoplasm. Pulmonary sarcoidosis in a 61 y.o. man affected by follicular lymphoma of the scalp. Axial (**a**) and coronal (**b**) computed tomography (CT) images show bilateral clusters of confluent micronodules (“galaxy sign”), preferentially located in the middle and superior zones of both lungs, with peribronchovascular and subpleural distribution

*Sarcoidosis* is commonly characterized on CT by well-defined, bilateral solid pulmonary micronodules (2–4 mm in diameter) with perilymphatic distribution; macronodules (≥ 5 mm in size) derived from the coalescence of micronodules may occasionally be observed [30]. In cancer patients, sarcoidosis may occur before, after, or concurrently with the diagnosis of tumour and an etiological relationship has been observed in at least 25% of cases where both conditions coexist [31]. This association may be due to the immune dysfunction and chronic inflammation that characterize sarcoidosis. Additionally, antineoplastic drugs may play a role in triggering the onset or exacerbation of sarcoidosis. Furthermore, chemo- and immunotherapeutic agents may cause *sarcoid-like reaction*, with development of noncaseating granulomas usually adjacent to the tumour/metastasis or in draining lymph nodes [31–34]. Some findings can be particularly useful for the differential diagnosis among pulmonary metastases and sarcoidosis. First, 75–95% of sarcoidosis patients have mediastinal and hilar lymphadenopathies, often with bilateral and symmetrical distribution, sometimes with egg-shell or inner punctuate calcifications. Additionally, sarcoid nodules typically demonstrate a perilymphatic distribution, particularly along the peribronchovascular interstitium and subpleural and perifissural areas. Sarcoid nodules generally predominate in the upper lobes and have a patchy distribution, and when confluent appear as a “galaxy” [35, 36] (Fig. 3). In the late stage of sarcoidosis, fibrotic changes can be found, such as reticular opacities, architectural distortion, traction bronchiectasis and volume loss, especially in the upper zones [30].

**Fig. 4:**
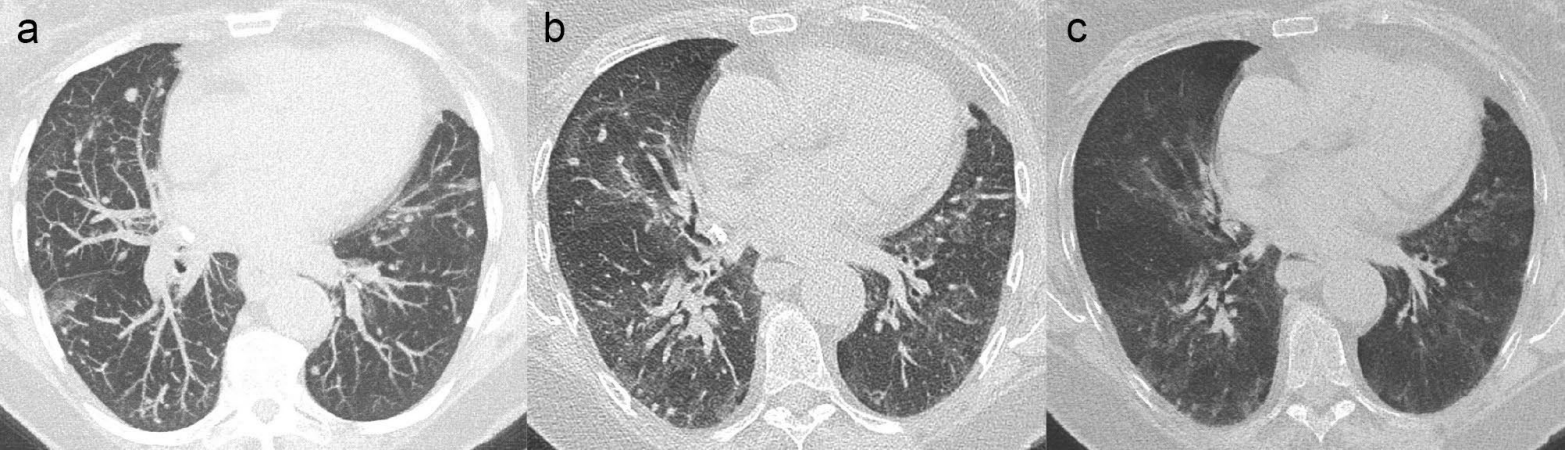
DIPNECH in an oncologic female patient. Diffuse idiopathic pulmonary neuroendocrine cell hyperplasia (DIPNECH) in a 63 y.o. woman who underwent gastric resection due to gastric adenocarcinoma. Axial computed tomography (CT) maximum intensity projection (MIP) image (**a**), expiratory low dose image (**b**) with minimum intensity projection (MinIP) reconstruction (**c**) showing well-defined bilateral nodules of various size and diffuse inhomogeneous density of lung parenchyma due to air trapping, better depicted in the expiratory images

*DIPNECH* is a rare condition characterized by abnormal proliferation of pulmonary neuroendocrine cells into the bronchial wall, being considered as a precursor of lung carcinoid tumours. DIPNECH usually affects middle-aged and elderly women, with no evidence of smoking correlation [37, 38]. It is characterized by well-defined solid (or less frequently ground-glass) bilateral pulmonary nodules of various size, spherical or ovoid, that represent either tumorlets (< 5 mm) or carcinoid tumours (≥ 5 mm). They typically show a peribronchiolar, lower-zone predominant and peripheral location. Due to cell proliferation within the bronchial walls, associated CT signs are bronchial wall thickening, mild bronchiectasis, mucoid impaction, and mosaic attenuation caused by multilobular air trapping [38, 39] (Fig. 4). These features are useful clues in discriminating between DIPNECH and pulmonary metastases in middle-aged women.

**Fig. 5:**
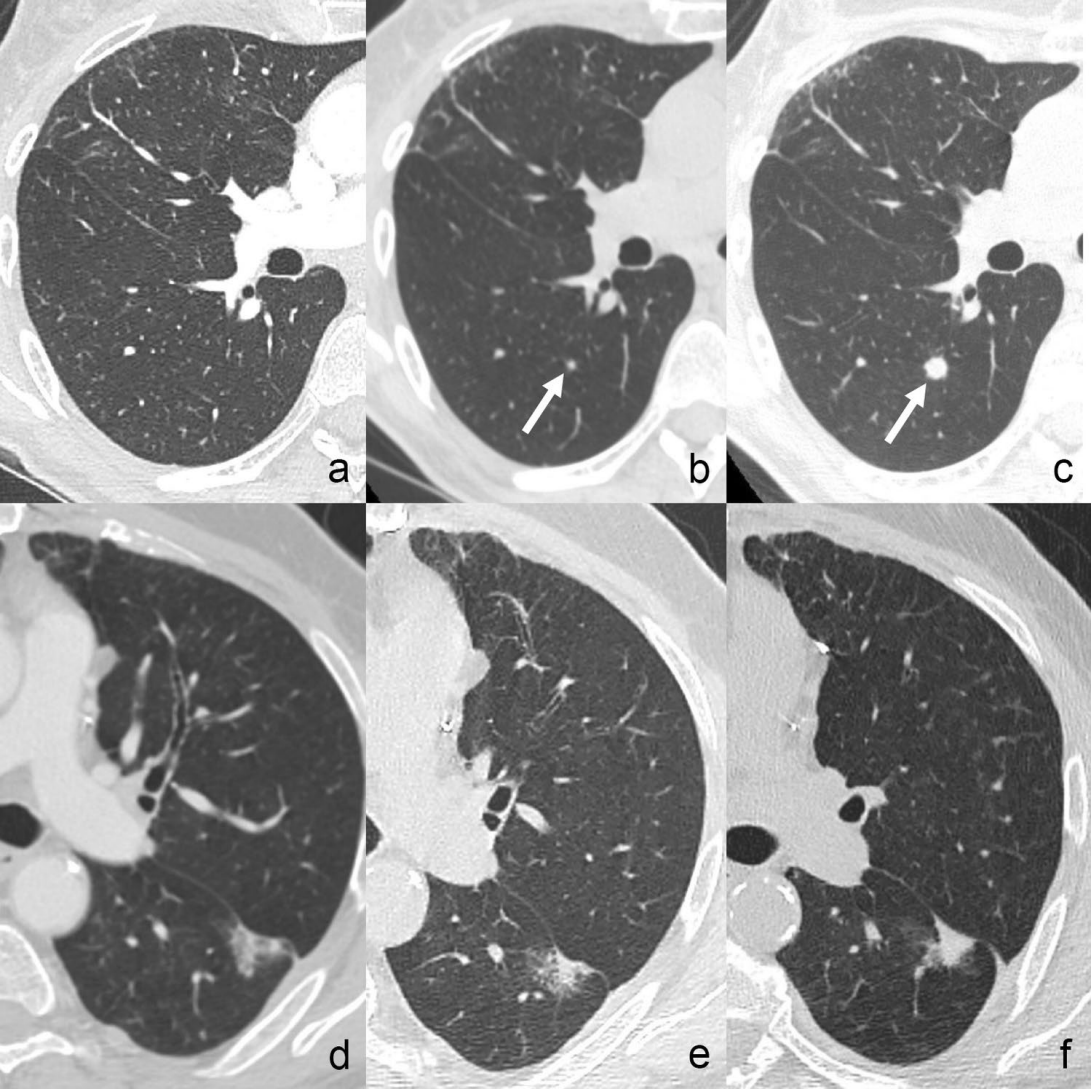
Organizing pneumonia versus lung adenocarcinoma, appearing as a solitary pulmonary nodule in two different cancer patients. **a**–**c** Solitary pulmonary nodule in a 50 y.o. woman appeared during the follow-up for breast cancer treated with surgery and radiotherapy. At first computed tomography (CT) exam **a** no pulmonary nodules are detected. Six months later **b** a tiny micronodule (arrows) can be observed in the superior segment of the right lower lobe. One year later **c** the nodule significantly increased in size and patient was addressed to surgery. Histology revealed a focus of organizing pneumonia. **d**–**f** Solitary pulmonary part-solid nodule in the superior segment of the left lower lobe in a 68 y.o. man with bladder cancer. A progressive increase of the solid component within the nodule, with retraction of the adjacent fissure, is observed during the follow-up, after 3 months (**b**) and 9 months (**c**). At surgery, histology revealed a lung adenocarcinoma

Approximately 2–10% of all *solitary pulmonary nodules* (SPNs) represent pulmonary metastases. In individuals without a previous history of cancer, the probability of SPN representing a metastasis ranges from 0.4 to 9%, while in oncologic patients this probability rises to 25% [16]. When a SPN is detected in a patient with ETNs, the likelihood of being a metastasis rather than a primary lung cancer mostly depends on patient’s age and smoking history [19]. Primary tumour histology has also a relevant role, considering that sarcoma, melanoma, and testicular cancer are more likely to cause solitary nodular metastasis than other malignancies [40]. Characteristics associated with increased likelihood of primary malignancy of a SPN are spiculated margins, upper lobe location, pleural indentation and/or retraction [40, 41] (Fig. 5). Moreover, the presence of isolated hilar and/or mediastinal nodal enlargement in the preferential site of lymphatic drainage according to the nodule location is highly suggestive of lung cancer [40, 42].

Table 1 highlights the main CT imaging features useful for the differential diagnosis among pulmonary metastatic nodules, nodular non-malignant pulmonary disorders which may resemble metastases, and primary lung cancer.

**Table 1:** Main computed tomography (CT) features of metastatic pulmonary nodules versus non-malignant nodular lung diseases and primary lung cancer

CT findings	Metastases [6, 11, 14–17, 38, 40, 46]	OP [20, 25–27, 46]	Sarcoidosis [28, 33, 46]	DIPNECH [35–37]	Lung cancer [11, 39, 40, 46]
Number	Variable, often multiple	Variable	Multiple, rarely solitary	Multiple	Generally solitary
Margins	Smooth/irregular/well-defined/ill-defined	Spiculated/irregular/lobulated/smooth/ill-defined	Generally ill-defined/confluent (well-defined in small discrete nodules)	Smooth/well-defined	Lobulated/irregular/spiculated
Shape	Round/oval/irregular	Round/oval/polygonal (especially in large lesions)	Irregular (rounded in small nodules)	Round/oval	Variable
Size	Variable and even heterogeneous	Variable	< 4 cm	Variable	Generally larger than metastasis
Density	Solid/ground glass/solid with surrounding ground glass (halo)/calcified/adipose	Solid/mixed density/ground glass	Solid	Solid/ground glass	Solid/part-solid/ground glass
Distribution	Usually bilateral and symmetrical	Generally bilateral with characteristic fluctuating and migratory behaviour during follow-up	Bilateral	Bilateral	Preference for right lung
*longitudinal*	Basal regions predominance	Randomly distributed	Predominance in upper and middle regions (sometimes widespread)	Lower regions predominance	More frequent in the upper lobes
*axial*	Peripheral regions predominance	Scattered, with predominance along bronchovascular bundles	Perilymphatic pattern (generally, perihilar and peripherally located)	Mainly peripheral, predominantly peribronchiolar, often within areas of air trapping	Either peripheral or centrally-located
Growth	Variable	–	–	Slow increase in size and number	Variable (depending on histology)
Associated imaging signs	Feeding vessel sign, *halo* sign	Open bronchus sign, *halo* sign, reverse *halo* sign	*Galaxy* sign, air bronchogram, *halo* sign, reverse *halo* sign	–	*Corona radiata* sign, *halo* sign
Mediastinal lymph nodes	Lymphadenopathies variably observed	Sometimes present (mostly lower paratracheal, subcarenal and hilar)	Mediastinal and hilar lymphadenopathies (75–95%), often bilateral and symmetrical; often calcified (generally not completely and with various pattern)	Possible mediastinal or hilar lymphadenopathies	Mediastinal and hilar lymphadenopathies frequently observed
Ancillary findings	–	Perilobular opacities, consolidation, ground glass opacity, parenchymal bands, bronchial wall-thickening and dilatation (generally in areas with extensive consolidation), fibrotic abnormalities	Irregular thickening of peribronchovascular interstitium, patchy ground glass opacities, patchy consolidation, air trapping, tracheobronchial abnormalities, fibrotic abnormalities	Extensive multilobular air trapping, mosaic attenuation, bronchial wall-thickening, mucoid impaction, bronchiectasis	Emphysema, bronchial wall-thickening, respiratory bronchiolitis, fibrotic abnormalities

CT, computed tomography; OP, organizing pneumonia; DIPNECH, diffuse hyperplasia of pulmonary neuroendocrine cells

### Miliary pattern

The *miliary pattern* consists of multiple, sharply defined and tiny (3–5 mm) nodules randomly distributed throughout the lungs, without any preferential location, due to hematogenous spread. This pattern of lung dissemination may be found in highly vascularized primary tumours, such as medullary thyroid carcinoma, melanoma, renal cell carcinoma, ovarian cancer, osteosarcoma, and choriocarcinoma [13].

**Fig. 6:**
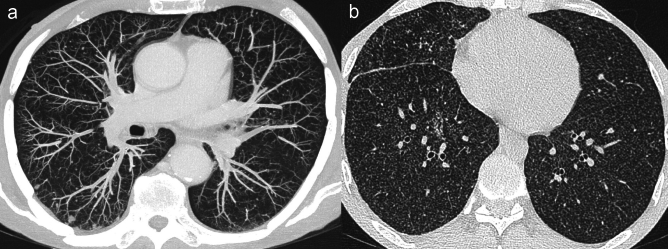
Miliary distribution of lung metastases versus miliary tuberculosis. **a** Miliary metastases in a 72 y.o. man with gastric carcinoid tumour endoscopically resected. Axial computed tomography (CT) maximum intensity projection (MIP) image shows multiple metastatic micronodules with a miliary distribution and variable size. Note the feeding vessel sign in the two larger nodules located in the right lower lobe. **b** Miliary tuberculosis in a 33 y.o. man. Axial CT scan depicts multiple micronodules widely disseminated throughout the lungs

In differential diagnosis we would consider *miliary tuberculosis,* which shows similar imaging findings, with both sharply and poorly defined micronodules widely disseminated within the lungs. Unlike metastases, in miliary tuberculosis all nodules tend to have the same dimension. When present, associated CT findings, such as calcified lung granulomas, tree-in-bud pattern, air trapping, pleural thickening and intrathoracic necrotic lymphadenopathies, are highly suggestive of the diagnosis of miliary tuberculosis rather than metastatic lung dissemination in cancer patients [43, 44] (Fig. 6).

### Cavitation and cysts

Cystic and cavitary pulmonary metastases most often occur in tumours of epithelial origin, especially squamous cell carcinoma (SCC) of the head and neck. However, cavitation has been described also in metastatic adenocarcinomas and sarcomas [45]. Cavitation is generated by tumoral inner necrosis or distal airspace dilation due to tumour infiltration into the bronchi, resulting in a check-valve mechanism. Additionally, chemotherapy can induce cavitation, indicating a partial response to treatment [45].

**Fig. 7:**
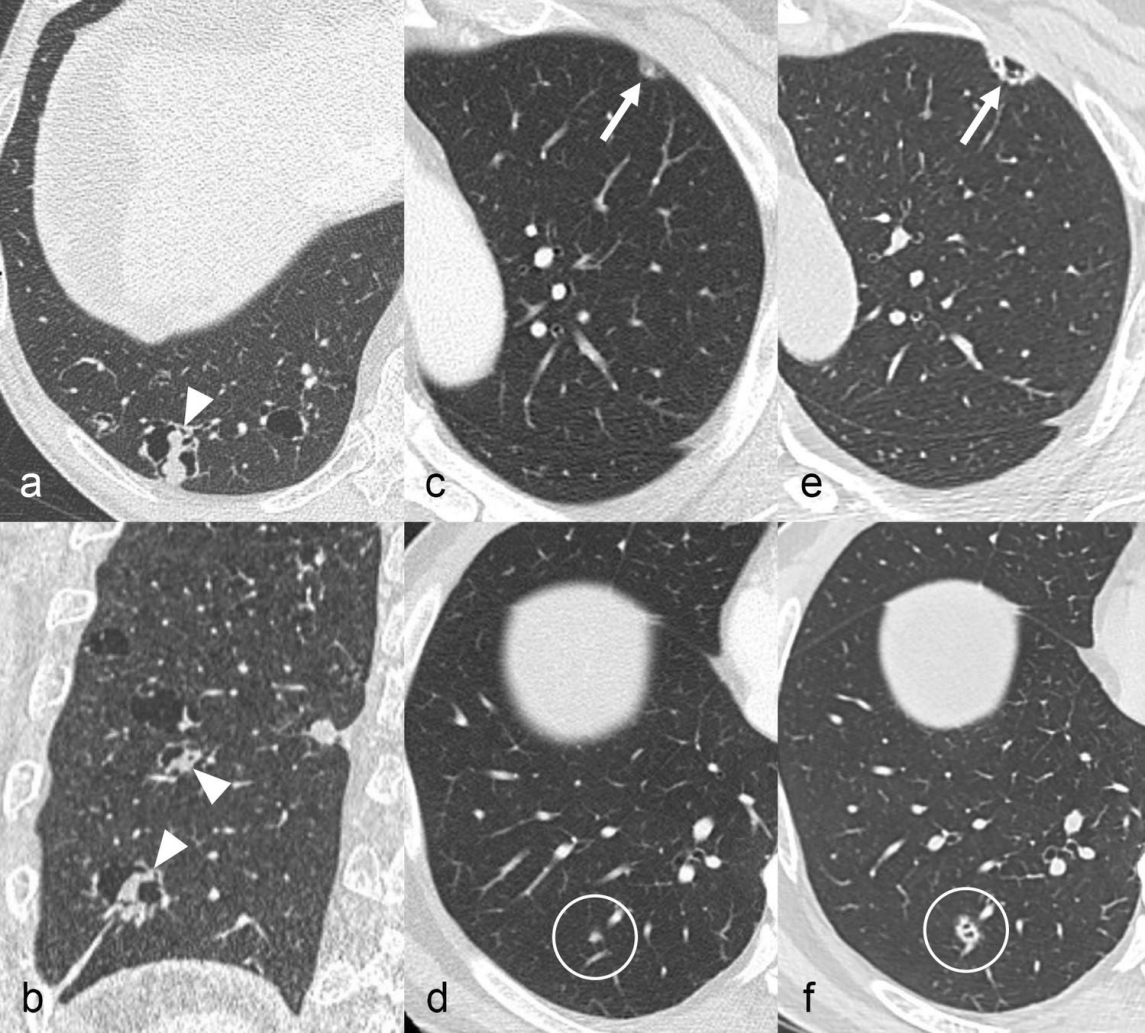
Cystic and cavitary pulmonary metastases. **a**, **b** Axial (**a**) and coronal (**b**) images in a 64 y.o. man with history of esophageal adenocarcinoma showing cystic lesions with thickened and lobulated wall, the majority with a solid and irregular component inside or at the periphery (arrowheads), histologically proved to be cystic metastases. **c**–**f** Cavitary pulmonary metastases in a 61 y.o. man affected by ampullary cancer. Axial computed tomography (CT) scans show two indeterminate subcentimeter nodules in the anterior segment of the left upper lobe (arrows) (**c**) and in the posterior segment of the right lower lobe (circles) (**d**), respectively. An increase in size and the appearance of small cavitation within both lesions at CT performed after 18 months suggested the hypothesis of metastases (**e**, **f**)

Cavitary metastatic nodules can be round or oval, with a thick and irregular wall, even though thin-walled cavities can be found [19] (Fig. 7). Usually, cystic lesions show a rounded shape with well-defined thin-wall (< 2 mm), whereas cavitary lesions demonstrate a thicker wall and a more irregular shape [46]. Pneumothorax can occur as a complication when a cavitary lesion communicates with the pleural space [45, 47].

Among other pulmonary disorders with cystic appearance, we should consider multifocal or diffuse diseases, such as *pulmonary Langerhans cell histiocytosis* (PLCH), a smoking-related disease that can mimic cystic metastases, particularly in the early/intermediate nodular phase of the disease. PLCH is characterized by multiple irregular, thick- or thin-walled cystic lesions (*cheerios* sign), that can be coalescent and variable in number and size, depending on the phase of disease [45, 48]. A combination of cysts and nodules is often observed [49]. Nodules show ill-defined margins and centrilobular distribution [50]. Differently from metastatic lesions, cysts are predominant in the upper-middle lung zones, generally symmetric, with sparing of the costophrenic angles [45].

### Calcifications

Pulmonary metastases may exhibit inner calcifications, as a result of necrotic phenomena, mucoid material deposition, intralesional bony degeneration, or as an intrinsic feature of the primary ETN [13]. Additionally, lung metastases can calcify as a response to chemotherapy [12]. Calcifications can demonstrate an amorphous, punctate, or reticular pattern [51]. The ETNs more often associated with calcified metastases are colorectal cancer (mucoid calcification), osteo- and chondrosarcoma (bone formation) and papillary thyroid carcinoma (dystrophic calcification) [13, 19].

**Fig. 8:**
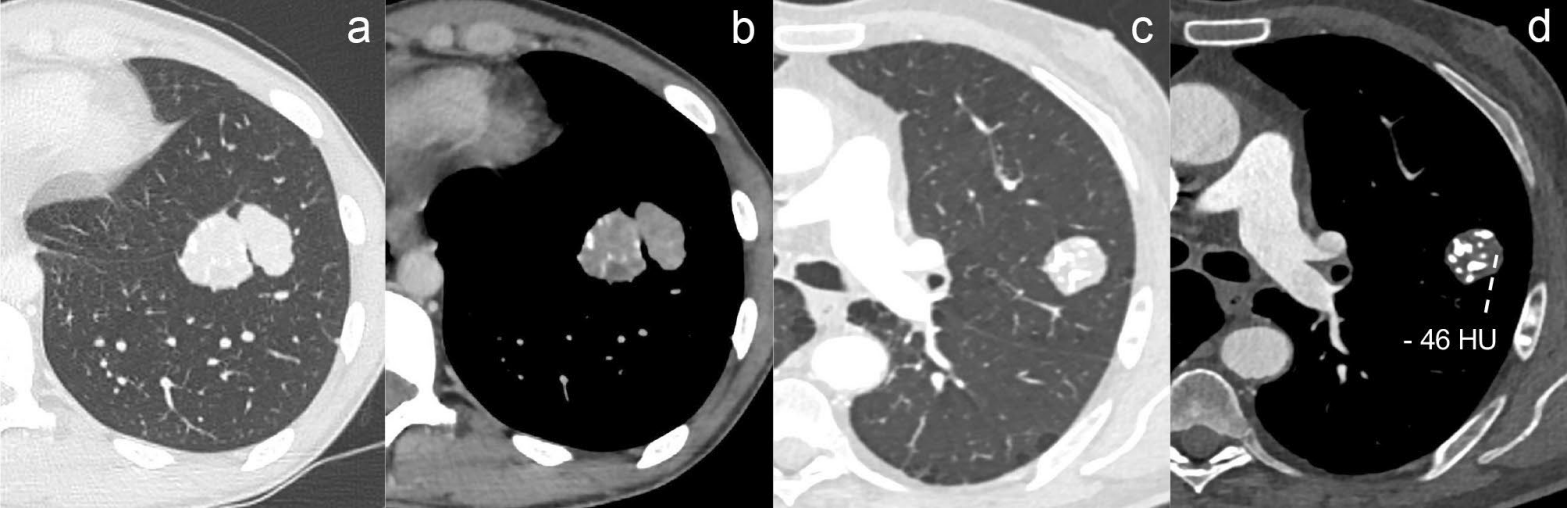
Pulmonary calcified metastases versus pulmonary calcified hamartoma. **a**, **b** Pulmonary calcified metastases in a 23 y.o. man affected by osteosarcoma. Contrast-enhanced axial computed tomography (CT) images showing two adjacent solid nodules with inhomogeneous contrast enhancement and eccentric irregular calcifications. **c**, **d** Pulmonary calcified hamartoma in a 73 y.o. man with a history of pancreatic adenocarcinoma, treated with pancreatectomy and adjuvant radio-chemotherapy. Axial CT images depict a nodule showing central dense calcifications with popcorn-like appearance; note the associated fat tissue density (-46 HU) within the nodule

Pulmonary calcifications can have a wide variety of non-neoplastic causes, and—among the others—environmental exposure should be taken into consideration in case of multiple nodules, while pulmonary hamartoma (PH) in case of a single lesion. PH can show calcifications in 15–30% of cases, commonly with a typical popcorn-like pattern [51, 52] (Fig. 8).

*Pneumoconiosis* encompasses a diverse array of diseases associated with exposure to dust, chemicals, or proteins [53]. A nodular pattern, characterized by small, circumscribed nodules (typically 2–5 mm large) with a perilymphatic distribution, predominantly located in the middle and upper posterior lung zones, can be observed mainly in patients affected by silicosis and coal miner’s pneumoconiosis, rather than other pneumoconiosis, such as asbestosis, berylliosis, or hard metal disease [54, 55]. Nodules may calcify, often diffusely, and be associated with egg-shell calcifications in hilar and mediastinal lymph nodes [53]. Additional findings useful for the differential diagnosis include pleural thickening and effusions, pseudoplaques, emphysema, and signs of fibrosis, with parenchymal distortion and mass-like consolidation [53].

**Fig. 9:**
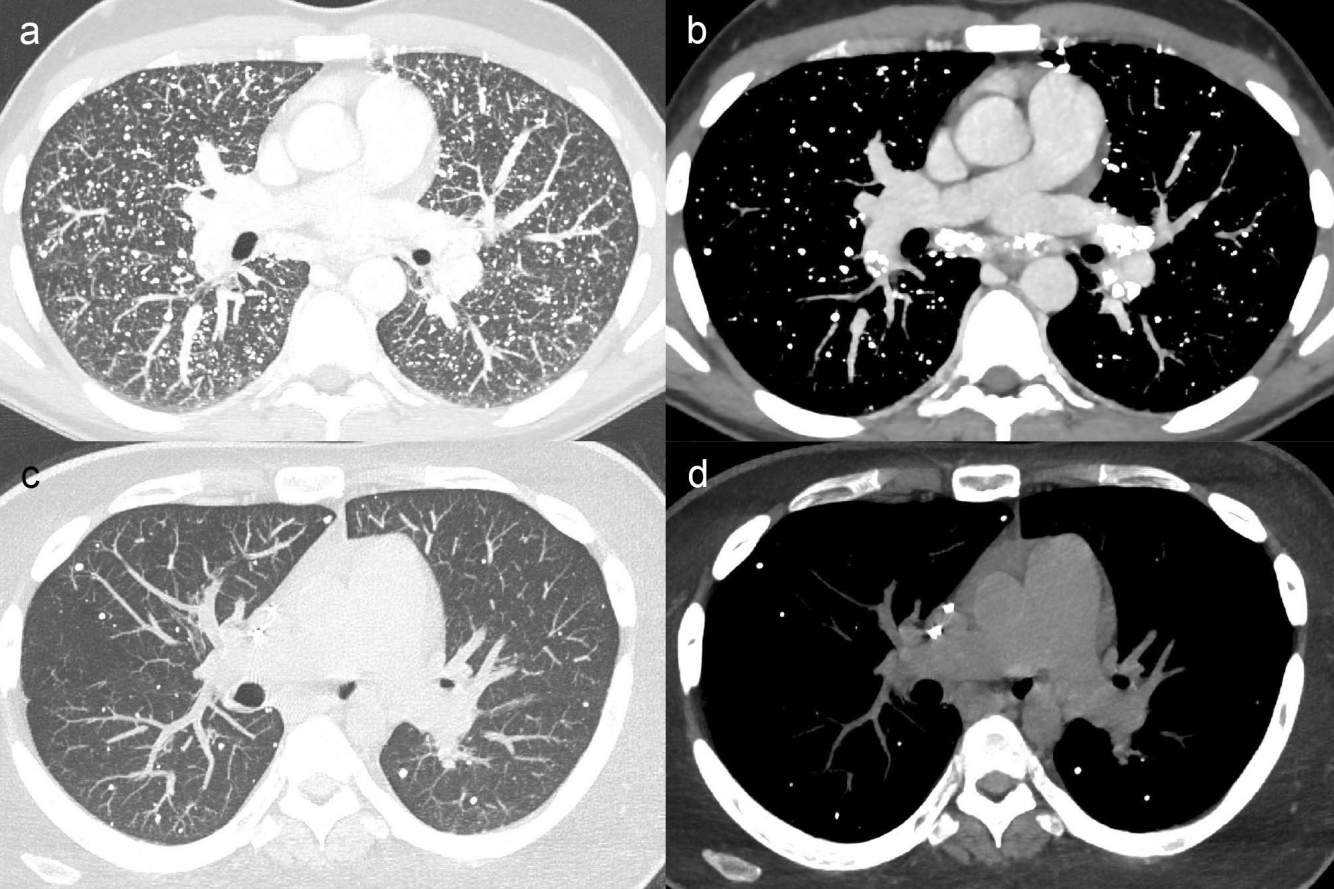
Calcified miliary metastases versus calcified nodules resulting from a previous infection. **a**, **b** Calcified metastases in a 33 y.o. man, who had undergone thyroidectomy due to a medullary thyroid cancer 10 months before. **a** Axial computed tomography (CT) maximum intensity projection (MIP) image depicts multiple sharply defined calcified micronodules with miliary distribution; note calcified hilar and mediastinal lymph nodes in **b**. Differential diagnosis with pneumoconiosis might be hard even though miliary distribution is less likely to be related to exposure, and patient’s age and history should always be taken into consideration. **c**, **d** Calcified nodules in a 38 y.o. man with previous varicella pneumonia. Axial CT MIP images show multiple calcified nodules and micronodules randomly distributed throughout the lungs, which are commonly found years after varicella infection

Diffusely distributed calcified pulmonary nodules and micronodules (< 5 mm in diameter) may also be a result of a previous infection (mostly mycobacterial, histoplasmosis, and varicella pneumonia), causing dystrophic calcification, which therefore must be included in the differential with calcified lung metastases [51, 52] (Fig. 9).

### Less common morphological features

Depending on the primary malignancy and its pattern of dissemination, pulmonary metastases can show a great variety of less common appearances on CT scans, such as halo sign, ground-glass, fat density nodules and neoplastic arterial embolism [19].

**Fig. 10:**
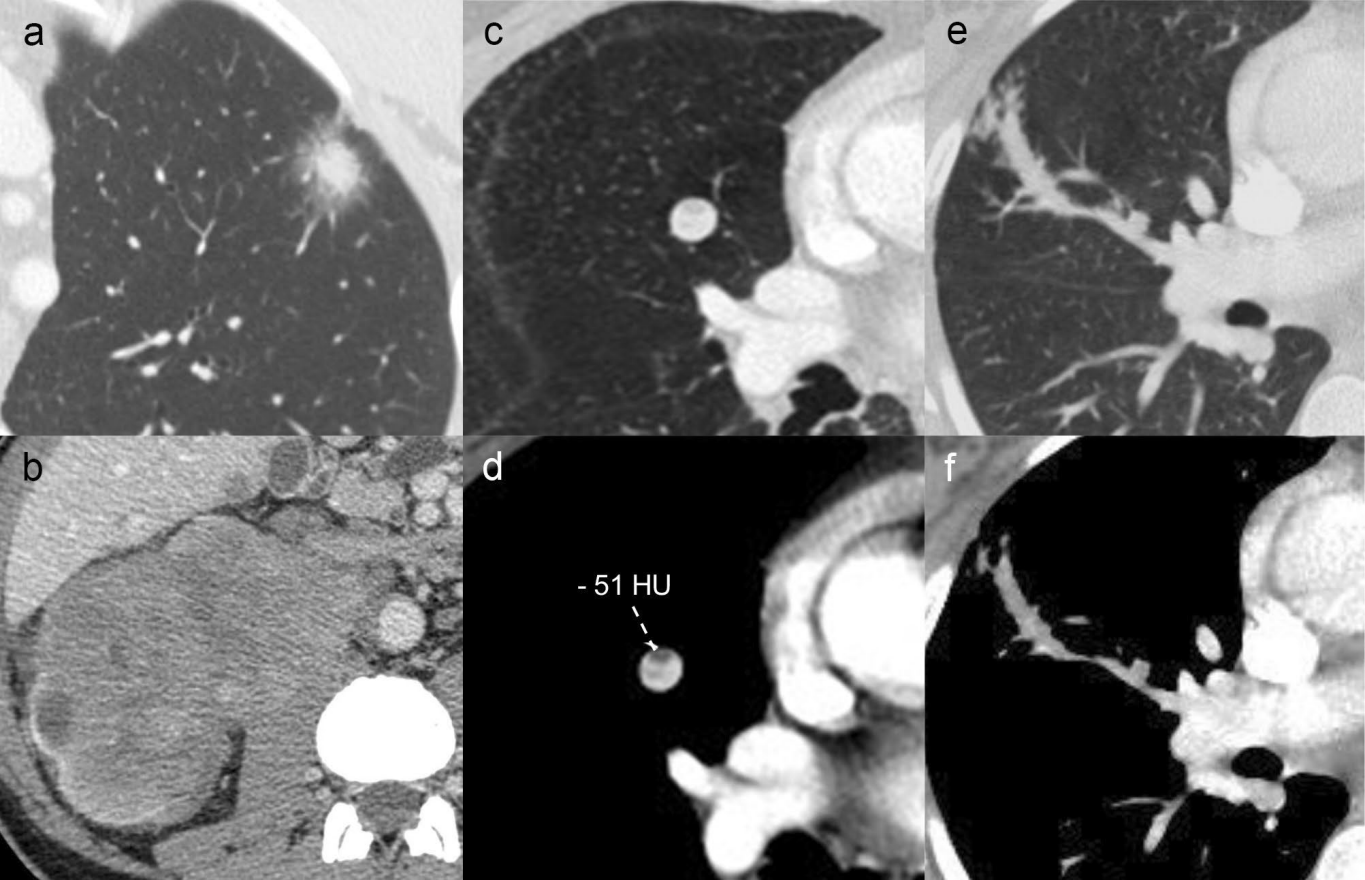
Uncommon CT features of pulmonary metastases. Metastases with less common morphological features. **a**, **b** Pulmonary metastasis in a 47 y.o. man with renal cell carcinoma. Axial computed tomography (CT) chest image **a** shows a pulmonary solid nodule surrounded by a peripheral ground-glass attenuation (halo sign) in the left upper lobe. Contrast-enhanced axial CT scan of the upper abdomen **b** depicts a large solid neoplastic mass of the right kidney, with marked heterogeneous contrast enhancement. **c**, **d** Pulmonary metastasis in a 78 y.o. man with renal cell carcinoma. Axial CT images depict a well-defined and contrast-enhancing pulmonary nodule; note the fat tissue density within the nodule in **d**. **e**, **f** Endoarterial tumoral embolization in a 61 y.o. man with gastric cancer. Axial CT images show vascular tree-in-bud pattern; note the irregular enlargement and occlusion of the arterial pulmonary vessels determined by intraluminal neoplastic tissue, evident until the centrilobular core

The *halo* sign is defined as a peripheral ground-glass attenuation surrounding a pulmonary nodule, as expression of peritumoral haemorrhage (Fig. 10). It is most commonly reported in pulmonary metastases from hypervascular tumours, such as angiosarcoma, choriocarcinoma, osteosarcoma and melanoma [56]. Nonetheless, a halo sign can be observed in non-haemorrhagic metastases (from adenocarcinomas and melanoma, among the others), resulting from peri-nodular lepidic growth, with tumoral cells spreading along intact alveolar walls [13, 18].

Metastatic lung tumour should always be suspected when, in a patient with history of an ETN, a rapid growth of multiple ground-glass and part-solid nodules occurs, with infection (atypical bacterial, mycotic, and viral infections) being the most common differential in these cases [57, 58].

Pulmonary metastases from renal cell carcinoma (RCC) and liposarcoma may rarely show macroscopic fat attenuation [59]. In case of a single metastasis from RCC (30%) [60], the evidence of inner fat attenuation (from − 40 to − 120 Hounsfield Units) on CT might represent a struggle for the differential diagnosis with PH. A significantly higher contrast enhancement within the nodule is more likely indicative of metastasis from RCC (Fig. 10) rather than PH. On the other hand, the coexistence of fat density (observed in 34 to 50% of PH cases) and central calcifications within the nodule is highly specific for PH [52] (Fig. 8).

Primary neoplasms such as hepatocellular carcinoma, breast and renal cell carcinomas, gastric and prostate cancers, and choriocarcinomas are associated with neoplastic endoarterial embolization. In rare cases, tumoral emboli can be visualized on CT within pulmonary arteries, with findings characterized by multifocal dilation of the peripheral subsegmental arteries, vascular tree-in-bud opacities, and peripheral wedge-shaped attenuation due to pulmonary infarction [18, 19] (Fig. 10). The most important differential diagnosis for this rare appearance is infectious bronchiolitis, which exhibits a tree-in-bud pattern due to bronchiolar inflammatory involvement. A clue for the differential is the presence of arterial enlargement in the vascular tree-in-bud pattern. Following the course of segmental and subsegmental arteries and bronchi with multiplanar and maximum intensity projection (MIP) reconstructions is crucial to differentiate these two patterns.

## Radiomics

The last decade witnessed a growing interest in the application of artificial intelligence (AI) algorithms for the evaluation of lung lesions, especially the radiomics method [61, 62]. Literature published so far has focused on primary pulmonary tumours, mainly on non-small cell lung cancer (NSCLC). The possibility of using radiomics in the characterization of lung metastases and in the differential diagnosis with primary tumours remains limited [63].

Kirienko et al. evaluated a cohort of 534 patients with pulmonary lesions, extracting textural features from positron emission tomography (PET) and CT images to differentiate between primary lung cancer and pulmonary metastases; the developed model exhibited a good performance for the CT dataset (area under the curve (AUC) of 0.79 and 0.70, for training and validation, respectively) and an excellent one for the PET (AUC of 0.92 and 0.91) [64]. Recently, a machine learning algorithm has been applied to predict the histotype of 445 pulmonary tumours in patients undergoing 18-Fluorine-Fluorodeoxyglucose Positron Emission Tomography/Computed Tomography (18F-FDG PET/CT); the combination of CT and PET features allowed a precise discrimination between primitive and secondary tumours (accuracy and AUC of 0.98) [65].

In patients with colorectal cancer and indeterminate pulmonary nodules (IPNs) at CT, a nomogram based on clinical-radiomics demonstrated a very good performance in discriminating between metastases and other lesions (AUC of 0.929 in the training dataset and 0.922 in the validation dataset), also achieving a satisfactory clinical utility according to the decision-curve analysis for almost all the threshold probabilities [66]. Such a model could provide useful indications for clinicians to choose among patients requiring further invasive procedures—including metastasectomy—and others who could be managed conservatively.

Furthermore, Zhong et al. developed a promising comprehensive clinico-radiological and radiomics model, which demonstrated a high accuracy in differentiating between pulmonary metastases and secondary primary lung cancers in patients with different neoplasms presenting solid pulmonary nodules at the baseline CT, reaching an AUC of 0.9421 and 0.9041 in the training and validation cohorts, respectively [67].

Despite the lack of standardization and external validation studies on large populations, which to date limits generalizability of the results, radiomics application on CT and PET images can potentially help in the characterization of lung metastases and in managing IPNs in patients with ETNs. These issues have been recently highlighted by Gabelloni and coauthors, which performed a systematic review on the role of radiomics in lung metastases. In fact, all the eight studies included were retrospective in design, only internally validated, and with data access not publicly available, demonstrating both a general poor quality (Radiomics Quality Score range: 22.2–38.9%) and a high variability, that poses a challenge in comparing the data and precludes a pooled analysis [63]. In the near future, external validation studies and clinical trials will be fundamental for establishing the feasibility of radiomics models in routine clinical practice.

## Management of pulmonary nodules in patients with ETNs

Due to the improvement in early cancer detection and treatment options, with the introduction of combined therapies and the employment of targeted effective drugs, cancer survivors have progressively increased [68, 69]. Concurrently, over the years, a dramatic increase in lung nodule detection rate has been observed due to the widespread use of CT imaging for medical purposes and technologic improvement in terms of spatial resolution of multidetector CT scanners [2, 70]. Although most incidental or IPNs are benign, their significance in patients with ETNs is controversial [71–77]. In cancer patients, nodules detected on chest CT may represent either a metastatic spread of disease, a benign process or even a second primary lung tumour [3]. CT can provide useful elements to assess the probability of nodule benignity or malignancy. Unfortunately, the majority of lung nodules detected on CT are small in size (diameter < 8 mm), therefore the recognition of morphologic features can be complex, so that they often remain indeterminate at imaging [78–80]. Furthermore, nodules are often too small (solid nodules or solid components < 8 mm) to allow metabolic characterization on 18F-FDG PET/CT scan or too small or located in complex site to obtain adequate tissue sampling [72, 77, 79, 81–83].

To date, no guidelines for the management and follow-up of pulmonary nodules in patients with ETNs exist. The most recent and widely applied international guidelines for pulmonary nodule management, in both clinical and screening settings, cannot be applied to this patients’ category [41, 84]. Also, the use of validated risk predictive models based on clinical and radiological nodule characteristics, as recommended by the British Thoracic Society (BTS) guidelines, still require further research and adequate validation in patients with known ETNs [85]. Indeed, a fairly recent survey among the Society of Thoracic Radiology (STR) members on the management of nodules incidentally detected in cancer patients showed that most radiologists still rely on their own “experience and common sense” and routinely recommend short-term CT follow-up primarily according to size, among nodule features, and to "interval since last normal imaging study", among clinical characteristics [86].

Over the years, several studies have dealt with pulmonary nodules in patients with ETNs, some of which grouping patients with different type of neoplasms, as summarized in Table 2, while others focusing on specific cancers, as summarized in Table 3. These studies reported variable rates of benignity and malignancy, providing predictive elements for malignancy [71, 72, 74–77, 81, 82, 87–99] and, in some cases, even indications for management and follow-up [71–77, 81, 82, 88–93, 96–99]. However, studies are not always fully comparable, due to the wide heterogeneity in technical aspects, inclusion criteria, and timing of follow-up.

**Table 2:** Main clinical studies published in the last two decades dealing with pulmonary nodules in patient populations with heterogeneous extra-thoracic neoplasms (ETNs)

Study	Population	Nodule features	Results	Indications for follow-up/management
Quint et al. (2000) [89]	- 149 pts with extrapulmonary cancer; total of 161 subject considered (patients with multiple primary cancers were considered multiple times) - 5 groups based on primary tumour: *Group 1*: squamous cell carcinoma of head and neck; *Group 2*: lymphoma or leukemia; *Group 3*: carcinomas of the bladder, prostate, breast, uterine cervix, ovary, esophagus, stomach, biliary tree; *Group 4*: carcinomas of the salivary glands, parotid glands, thyroid, thymus, adrenal gland, kidney, colon, uterus; *Group 5*: melanoma, sarcoma, testicular cancer	- SPNs; - diameter: ≥ 5 mm; - revealed at a CT scan performed most commonly for follow-up or as further investigation for CXR abnormalities	*Group 1*: 33 pts PLC: 25 (76%); Metastasis: 3 (9%); Benign lesion: 5 (15%) *Group 2*: 14 pts PLC: 8 (57%); Metastasis: none; Benign lesion: 6 (43%) *Group 3*: 45 pts PLC: 26 (58%); Metastasis: 8 (18%); Benign lesion: 11 (24%) *Group 4*: 31 pts PLC: 13 (42%); Metastasis: 16 (52%); Benign lesion: 2 (6%) *Group 5*: 38 pts PLC: 9 (24%); Metastasis: 23 (60%); Benign lesion: 6 (16%) Correlation of SPN histology with: 1. *Primitive tumour*: - In *groups 1* and *3* higher risk of PLC rather than metastasis (ratio 25:3 and 26:8, respectively); - *Group 4* almost overlapping odds (ratio 13:16); - *Group* 5 greater risk of metastasis rather than PLC (ratio 23:9) 2. *Smoking history:* smokers have a 3.5-times higher risk of developing lung cancer than non-smokers	None
Khokhar et al. (2006) [90]	- 151 pts with history of extrapulmonary neoplasia - Pts divided into 4 groups with increasing probability of lung metastasis, excluding hematologic neoplasms: *Group 1*: carcinomas of head and neck; *Group 2*: carcinomas of the esophagus, breast, ovary, cervix, prostate and bladder; *Group 3*: carcinomas of the liver, colon, rectum, adrenal, kidney, uterus, and carcinoid; *Group 4*: melanoma, sarcoma, Kaposi’s sarcoma, testicular cancer and thymoma	- Both single and multiple nodules; - No size restrictions; - Mean size: 12 mm (range 6–20 mm)	- SPNs: 59/151pts (39.1%) (53% malignant, the majority PLC); - Multiple nodules: 92/151 pts (60.9%) (36% malignant; 39% PLC and 57% metastases); - Malignant nodules: 64/151 pts (42%): *PLC*: 32/64 (50%); *Metastases*: 28/64 (43.8%); *New cancers or undetermined etiology*: 6.2% (4/64) *Group 1*: higher rate of malignant nodules, greater prevalence of PLC rather than metastases (ratio 6:2); *Group 4*: similar amount of PLC and metastases (ratio 4:5) - Probability of malignancy associated with: smoking history (pack/years), nodule size, SPN and primary cancer in univariate analysis; smoking history (pack/years) and nodule size in multivariate analysis; - Smokers were more likely to have PLC	Need for close follow-up and requirement for diagnostic biopsy earlier compared to established malignancy risk threshold, particularly in patients with a history of smoking and with large nodules
Munden et al. (2010) [83]	- 102 pts; - Pts divided into two groups according to follow-up: *Group 1*: follow-up with CT ≤ 365 days; *Group 2*: follow-up with CT > 365 days	- Both single and multiple nodules; - Diameter ≤ 4 mm on initial staging CT	*Group 1*: 17 (36%): nodules increased in size; 9 (19%): nodules increased in size and number; 19 (40%): stable; 1 stable with new nodules; 1 decreased in size; - Mean time to increase: 203 days (63–355 days); average size increase of 5 mm (1–13 mm) *Group 2*: 3 (5%): nodules increased in size; 51 (93%): stable; 1: stable with new nodules; - Mean time to increase: 527 days (413–730); average size increase of 6.3 mm (5–7 mm) Increase in nodule size (suggesting malignancy) in 28% pts with 90% of nodules increased within the 1st year	- Follow-up at 3 months in the 1st year after pulmonary nodule detection; - In case of stability at 1 year follow-up, follow-up at 6 months for the 2nd year
Hanamiya et al. (2012) [91]	- 233 pts with extrapulmonary neoplasms; - 137/233 pts with nodules that met criteria for nodule malignancy or benignity; - Indetermined nodules excluded; pts divided into 4 groups with increasing probability of lung metastasis, excluding hematologic neoplasms: *Group 1*: squamous cell cancers of the head and neck; *Group 2*: carcinomas of the esophagus, stomach, biliary tree, urinary bladder, breast, ovary, uterine cervix, or prostate; *Group 3:* carcinomas of the salivary glands, parotid gland, thyroid gland, thymus, adrenal gland, colon, kidney, or uterus; *Group 4*: melanoma, sarcoma, testicular carcinoma - 3 groups based on nodule size: < 5 mm; 5–10 mm; ≥ 10 mm - 3 groups based on the distance from the pleura: < 5 mm; 5–10 mm; ≥ 10 mm	- Both single and multiple nodules; - Mean size: 7.4 mm (range 2–49 mm); - Mean distance from the pleura: 9.3 mm (range 2–51 mm)	- Single nodule: 48/233 pts (21%); - Multiple nodules: 185/233 pts (79%); - Malignant nodules: 28/137 (20.4%) (mean size: 17 mm; mean distance from pleura: 18.4 mm); - Benign nodules: 109/137 (79.6%) (mean size: 6.4 mm; mean distance from the pleura: 8.3 mm) *Group 1*: 6 pts Benign lesions: 4 (67%%); Malignant lesions: 2 (33%) *Group 2*: 77 pts Benign lesions: 66 (86%); Malignant lesions: 11 (14%) *Group 3*: 48 pts Benign lesions: 38 (79%); Malignant lesions:10 (21%) *Group 4*: 6 pts Benign lesions: 1 (17%); Malignant lesions: 5 (83%) - Highest probability of malignancy: > 10 mm in size (85%); ≥ 10 mm far from the pleura (47%); *Group 4* - On multivariable analysis, nodule size and distance from the pleura significantly predictive of malignancy (*p* < 0.0001 and *p* = 0.021, respectively)	Nodules < 10 mm in size and < 10 mm from the pleura can be followed with similar intervals to CT screening for person without cancer
Caparica et al. (2016) [92]	228 pts with extrapulmonary solid tumour	- Both single and multiple nodules; - Nodules ≥ 5 mm	- Single nodule: 112/228 pts (49.1%); - Multiple nodules: 116/228 pts (50.9%); *- Metastases*: 146/228 pts (64%); *- PLC*: 60/228 (26.3%); *- Benign*: 22/228 (9.6%) Multiple pulmonary nodules (> 5 mm) (OR = 5.08) and cavitation/necrosis (OR = 2.9) significant associated with metastasis at multivariate analysis	Biopsy is recommended for an accurate diagnosis and to avoid overestimation of metastatic nodules in cancer pts
Yang et al. (2017) [93]	- 161 pts with extrapulmonary neoplasms; - Nodules classified into:*stable* (change in size absent or ≤ 1–1.5 mm, no change in density); *changed* (disappearance, increase and/or reduction in size ≥ 1–1.5 mm, change in density)	- Both single and multiple nodules; - Size ≤ 10 mm at baseline CT	- 36.6% of nodules showed interval change at follow-up; - Average interval to the first change in size: 65 days (range 29–144 days); - Higher probability of nodule change at follow-up (malignancy): multiple nodules, smooth/slightly lobulated margins, advanced stage of malignancy (III e IV), no spiculation	Recommended follow-up at 1–3 months after nodule detection
de Morais et al. (2020) [94]	- 100 pts with extrapulmonary solid tumour; - 251 pulmonary nodules	- Nodules < 10 mm; - Mean size: 4.8 mm	- Stable nodules: 175/251 (69.7%); - Increased in size: 50/251 (19.9%); - Decreased in size: 20/251 (8%); - Increased in size, then decreased: 6/251 (2.4%) 1. Among nodules increased in size: - 28/50 (56%) showed growth within 3–6 month; - 3/50 (6%) showed growth within 3 months; - 19/50 (38%) showed growth after 6 months 2. Among nodules biopsied or surgically resected proved to be metastases (11/12): - 10/11 (90.9%) showed growth; - 1/11 (9.1%) was stable 3. Growth (risk of malignancy) associated with: - Male sex; - Advanced stage of disease; - Colorectal cancer; - Irregular, lobulated or spiculated margins; compared to regular margins	Early CT follow-up in the 1st year after diagnosis

ETNs, extra-thoracic neoplasms; Pts, patients; SPN(s), solitary pulmonary nodule(s); CT, Computed Tomography; CXR, chest X-ray; PLC, primary lung cancer; yrs, years; OR, odds ratio

**Table 3:** Most relevant clinical studies and systematic reviews on pulmonary nodules in patient populations with specific extra-thoracic neoplasms (ETNs)

Study	Primary tumour/population	Nodule features	Results	Indications for follow-up/management
Nordholm-Carstensen et al. (2013) [75]	- Colorectal cancer; - Systematic review of 12 studies; - 5873 pts; - 732/5873 pts (9%) with IPNs at preoperative staging CT - Follow-up performed with: CT, PET or PET/CT scan, invasive procedure, or not specified	- No restriction to specific definition of IPN - IPNs detected at staging CT	- IPNs: 732/5873 of pts (9%); - Metastases during follow-up in 10.8% of pts with IPNs 1. A meta-analytical assessment of the malignant potential of IPNs was not feasible due to heterogeneity and inconsistency issues; 2. Positive nodal status associated with malignant progression of IPNs (5 studies); 3. Number of IPNs associated with malignancy (3 studies); 4. Calcified IPNs associated with benign nature/irregular margin associated with malignancy (2 studies); 5. An association with malignancy was found with size ≥ 5 mm, central location, rectal cancer, presence of other distant metastases (3 studies overall)	- Presence of IPNs should not delay surgery with curative intent; - No evidence for additional diagnostic testing required for IPNs beyond routine follow-up
Xu et al. (2014) [74]	- Renal cell carcinoma (stages I–III); - 240 pts; - 92 pts with pulmonary nodules	- Nodules detected at baseline CT or CXR; - Average nodule number: 1.63 (range: 1–4); - Average nodule size: 0.46 cm (range: 0.1–2.3 cm)	1. Among pts undergoing CT, IPNs found in 88/170 (51.8%); 2. DFS significantly associated with (multivariate analysis): presence of lung nodules (negative effect), stage and grade (negative effect); 3. OS significantly associated with Charlson comorbidity score and primary tumour size (multivariate analysis), not with presence of pulmonary nodules; 4. Exclusion of patients with CXR did not result in significant changes in observed outcomes	Close surveillance after surgery in patients with IPNs on baseline chest imaging
Mano et al. (2015) [95]	- Renal cell carcinoma; - 748 pts; - 382 pts (51%) with IPNs on preoperative CT	- IPNs ≤ 20 mm; - Both single and multiple nodules; - Follow-up with CT for patients with IPNs and with CRX for patients without IPNs	1. No association between presence of IPNs and distant metastases or death from RCC; 2. Higher risk of metastastatic disease if nodule > 10 mm, compared to IPNs ≤ 10 mm, after adjusting for tumour histology, stage, and size; 3. Nodule size significant predictor of any distant metastases at multivariable analysis; 4. pts developing pulmonary metastases at 5 yrs: - With IPNs 1–2 cm: 27%; - With IPNs ≤ 10 mm: 10%; - No nodules: 9% 5. In 25/33 pts (76%) lung metastases originated from preoperative IPNs	- Extensive postoperative follow-up for patients with IPN(s) > 10 mm; - Detection of IPN(s) ≤ 10 mm should not delay curative treatment nor promote more intense follow-up protocol
Li et al. (2016) [96]	- Breast cancer; - 1325 pts; - 812 pts (61%) with at least one non-calcified nodule	- Single or multiple solid nodules detected on CT acquired from < 1 yr prior to > 1 yr after cancer diagnosis; - Nodule size range: 2–49 mm; - Subgroups based on size: *small*: < 10 mm; *large*: ≥ 10 mm	*- Benign nodules*: 482/812 pts (59.4%); *- Malignant nodules*: 330/812 pts (40.6%); 1. Metastases from breast cancer: 212/812 pts (26.1%); 2. Metastases from other cancers: 32/812 pts (3.9%); 3. PLC: 26/812 pts (3.2%); 4. Indeterminate or suspicious lesions: 60/812 pts (7.4%) - In pts with multiple large nodules malignancy rate of 83% (most of these being metastases); for very small nodules (2–4 mm), malignancy rate of 8% for solitary nodules and 20% for multiple nodules; in pts with solitary nodules, 46% had PLC and 12% metastases from breast cancer - Risk factors for malignancy: 1. Size (large > small nodules); 2. Number (multiple > solitary nodules); 3. Grade (lung metastases more frequent with grade 3 compared to 1–2); 4. Clinical stage (lung metastases more frequent in stage II–III than 0-I)	None
Hammer et al. (2017) [72]	- Breast cancer; - 211 pts; - 96 pts (45.5%) with nodules; - 248 nodules	- Both single and multiple nodules; - Detected on baseline CT	*- Benign nodules*: 84/96 pts (88%); *- Metastases*: 9/96 pts (9%); *- Other malignancy:* 3/96 pts (3%) (*PLCs:* 2/3 pts; *metastases from another cancer:* 1/3 pts) 1. Imaging predictors of malignancy: irregular or spiculated margins, lobulated shape, size of the largest nodule (malignant nodules larger than benign ones, with a median of 7 mm vs 4 mm), number of nodules, pleural studding, hilar lymphadenopathy, presence of extrapulmonary metastasis 2. Predictors of malignancy at multivariate analysis: largest nodule diameter, extrapulmonary metastases, nodules number	Decision-tree model to predict nodule benignity/malignancy (86% accurate overall per nodule; 91% accurate overall per patient), based on: size of the largest nodule, number of nodules, nodule shape, presence of pleural studding (indicative of pleural metastases), and presence of extrapulmonary metastases; additional factor: hilar lymphadenopathies
Soliman et al. (2019) [82]	- Melanoma; - 148 pts; - 70 pts with IPNs detected at baseline (among these, 11 subsequently developed other IPNs at follow-up); - 17 pts with IPNs detected at follow-up (interval IPNs)	Solid or subsolid nodules < 10 mm detected on baseline CT or during follow-up	1. At baseline: - IPN(s): 70/148 of pts (47.3%); - No nodule: 78/148 (52.7%); - Total of 243 nodules at baseline (metastases: 2/243; 0.8%) 2. At follow-up: - 40 IPNs developed in 28 pts - *Metastases*: 35/40 (87.5%) (in 25 pts) - *Benign*: 5/40 (12.5%) 3. Independent predictor of metastasis: interval nodule, volume change at 3 months, initial volume/diameter, distance from the pleura, combined stage IIC + III at diagnosis 4. 43% pts with IPNs proved to have metastases in 3 months; 72% in 6 months; 94% in 12 months	- Importance of 3-month follow-up and subsequent follow-up in 6 months for confirmation of nodule etiology; - Short-term follow-up also for perifissural nodules (11% of metastatic interval IPNs in perifissural sites)
Al-daghmin et al. (2019) [77]	- Bladder cancer; - 168 pts; - 74 pts (44%) with IPNs	- IPNs ≤ 3 cm on preoperative imaging; - Single or multiple nodules	1. Distant metastases at follow-up: 57/168 of pts (33.9%) 2. Risk factors for metastasis (multivariate analysis): presence of IPNs, nodule size > 10 mm, stage 3. Factors associated with DFS: presence of IPNs, size > 10 mm 4. No correlation between IPNs (presence, number and size) and OS; 5. Surgery significantly associated with better OS	Tailored follow-up after surgery and histological characterization of nodules > 10 mm, with possible early beginning of chemotherapy
Tsoi et al. (2021) [76]	- High-grade osteosarcoma/spindle cell sarcoma of bone; - 431 pts; - 68/431 pts (16%) with IPNs at baseline	IPNs < 10 mm	- IPNs at baseline: 68/431 of pts (16%); - Metastases developed in 50/68 of pts (74%) (median of 5.3 months); - 43/50 pts (86%) with disease progression within 2 yrs of diagnosis 1. IPNs associated with poorer OS and higher incidence of metastases; 2. Pts with baseline IPNs had worse metastasis-free survival at 2 years (35% vs 77% of pts with normal staging CT); 3. No association found between radiological features and OS	- CT follow-up for at least 2 yrs; - Considering IPNs in treatment decisions
Kuan et al. (2021) [73]	- Pancreatic adenocarcinoma; - Systematic review of 5 studies; - 763 pts with IPNs	Median nodule size: 0.5–0.65 mm	- IPNs at staging CT: 18–71%; - IPNs proved to be metastases: 1.5–16% 1. No significant differences in OS between patients with and without IPNs; 2. Due to limited data available, no potential associations between nodule size, number and characteristics and probability of malignancy were found; 3. A meta-analytical assessment was not feasible due to heterogeneity among studies and inconsistency of data	- Presence of IPNs should not preclude an intervention with curative intent; - Importance of postoperative surveillance given the possibility of early identification of IPNs progression, so that a proportion of patients can undergo potentially curative treatment
van den Broek et al. (2021) [78]	- Colorectal cancer; - Systematic review and pooled analysis of 18 studies; - 8637 pts; - 1217 pts with adequate follow-up data for outcome analysis on IPNs	No restriction to specific definition of IPN	- IPNs at staging CT: 1327/8637 pts (15.4%); - IPNs proved to be metastases at follow-up: 203/1217 pts (16.7%) 1. Heterogeneity of the included studies and lack of comparative arms precluded a meta-analytical assessment; 2. Predictive factors of malignancy (also derived from studies excluded from quantitative analysis): regional lymph node metastases, liver metastases, rectal cancer, larger IPN size (> 5 mm), multiple IPNs (> 1 or > 3); less frequently reported parameters: advanced T-stage, perineural invasion, bilateral IPNs, irregular margin of IPN, central IPN location, CEA elevation	- CT scan at 3–6 months after initial staging; - Follow-up length of 2 yrs; - If nodule > 10 mm an 18F-FDG PET/CT scan is advocated and, based on nodule location, biopsy or surgical resection should be considered
Brookes MJ et al. (2023) [101]	- High-grade soft tissue sarcomas; - 389 pts; - 134/389 pts (34.4%) with IPNs at staging CT	IPNs < 10 mm, non-calcified	- 27/134 (20.1%) IPNs progressed to metastasis; - Median time to progression: 143 days - Risk factors for IPN progression at univariate analysis: multiple IPNs, IPNs ≥ 5 mm, bilateral IPNs, primary ≥ 5 cm, primary grade 3, primary arising deep to the fascia; - Risk factors for IPN progression at multivariate analysis: IPNs ≥ 5 mm, primary grade 3	CT follow-up in pts with IPNs at 6 and 12 months
Chen et al. (2023) [100]	- Resected esophageal carcinoma; - 816 pts	New nodules at postoperative CT scans	- IPNs in 221/816 pts (27.1%); - Metastases in 66/221 nodules (29.9%); - Mean time of detection 22.86 months in the pulmonary metastasis group versus 11.44 months in non-pulmonary metastasis group; - Metastasis predominantly solid nodules (90.1%); non-metastatic nodules more frequently subsolid (62.3%); - Probability of pulmonary metastasis associated with: pathologic N category, size of the largest IPN, time of detection of IPNs, number of IPNs, and shape of the largest IPN at multivariate analysis	Pulmonary Metastasis Prediction Model scale (0–15 points) (AUC = 0.946): - Very low risk (0–6): 0.8%; - Intermediate risk (7–9): 25.7%; - High risk (10–15): 91.8%
Müller et al. (2023) [102]	- Head and neck SSC; - Systematic review of 24 studies; - 1667 pts	Excluded studies that examined only metachronous lung nodules, or only metachronous PLC, and studies where metachronous or synchronous nodules and second PLC were not analysed separately	- Synchronous lung nodules in mean 11.4% of pts; synchronous PLC in mean 2.95% of pts; possibility of a synchronous lung nodules to be a synchronous PLC in mean 35.2%; - 98–100% of pts with synchronous PLC were previous or active smokers; - 18F-FDG PET/CT is the most reliable method to diagnose synchronous PLC (sensitivity: 95%; specificity: 96%; PPV: 80%) - Detection of human papillomavirus, p16 immunohistochemistry, and clonal evolution useful in differentiating metastasis from synchronous PLC; - 5-year OS in case of synchronous PLC: 34–47%	None
Hassan et al. (2024) [99]	- Primary bone sarcoma; - Systematic review and pooled analysis of 6 studies (4 on osteosarcoma, 1 on chondrosarcoma; 1 on Ewing sarcoma); - 1667 pts (1054 with osteosarcoma, 440 with chondrosarcoma, 173 patients with Ewing sarcoma)	No restriction to specific definition of IPN, variable among the different studies	- IPNs at staging CT: 302/1667 pts (18.1%); - IPNs proved to be metastases at follow-up: 136/302 (45.0%); - Median time from baseline to metastases development: 7.9 months (according to 4 out of 6 studies) 1. Heterogeneity of the included studies precluded a meta-analytical assessment; 2. Variably reported predictive factors of malignancy: size > 5 mm, lobulated margins, bilateral IPNs; centrally or centrally and peripherally located nodules; partially calcified nodules 3. Owing the inconsistency in literature, prognostic impact of IPNs remains unclear	- CT scan at 3, 6 and 12 months after initial staging; - Follow-up length of minimum 2 yrs; - Follow-up especially required for IPNs > 5 mm in average diameter
Nuijens BW et al. (2024) [98]	- Colorectal cancer; - 204 pts with IPNs at baseline CT	Exclusion criteria: obvious lung metastasis, immediate palliative care, other malignancies in previous 5 yrs	- Temporal split for derivation (159/204) and validation sample (45/204): *Derivation sample*: 42/159 (26.4%) IPNs progress to metastases within 2 yrs; median time from preoperative CT to progression: 10 months; *Validation sample*: 12/45 (26.7%) IPNs progress to metastases within 2 yrs; median time from preoperative CT to progression: 8 months; - Risk factors of IPN progression: rectal location of primary tumour, pN2, extrapulmonary synchronous metastases at-diagnosis, size of largest nodule at baseline CT > 10 mm	- Nomogram model with percentage risk of IPNs of being metastasis (training set: AUC 0.83; validation set: AUC 0.79); - Follow-up might be extended from 3 to 6 months in low-risk cases, while being more frequent in high-risk ones
Cignoli D et al. (2024) [97]	- Resected renal cancer; - 121 pts, developing either lung metastasis or metachronous PLC during follow-up - Lung metastasis in 105/121 pts (86.8%), metachronous PLC in 16/121pts (3.2%)	Excluded pts developing metastases in multiple sites	- Risk factors that a pulmonary lesion detected during follow-up is a metastasis (univariate analysis): larger primary lesion size, higher clinical and pathological stage, higher grade, presence of necrosis and lymphovascular invasion, multiple nodules, bilateral nodules; - Risk factors that a pulmonary lesion detected during follow-up is a metachronous PLC (univariate analysis): history of smoking, longer interval between surgery and lung lesion detection; - Median PFS of 10.9 yrs for metachronous PLC and 3.8 yrs for lung metastasis; - Median OS 6.5 yrs for metachronous PLC and 6 yrs for lung metastasis	None

ETNs, extra-thoracic neoplasms; Pts, patients; IPN(s), indeterminate pulmonary nodule(s); CT, Computed Tomography; PET, Positron Emission Tomography; PET/CT, Positron Emission Tomography/Computed Tomography; CXR, chest X-ray; DFS, disease-free survival; OS, overall survival; RCC, renal cancer carcinoma; yr(s), year(s); PLC(s), primary lung cancer(s); CEA, carcinoembryonic antigen; 18F-FDG PET/CT, 18-Fluorine-fluorodeoxyglucose Positron Emission Tomography/Computed Tomography; AUC, area under the curve; SCC, squamous cell carcinoma; PLC, primary lung cancer; PPV, positive predictive value; PFS, progression-free survival; FDG-PET/CT, Fluorodeoxyglucose Positron Emission Tomography/Computed Tomography

Several factors showed a significant association with the likelihood of malignancy, as follows: history of cigarette smoking (significantly related to the development of lung cancer); primary cancer histology (melanomas, sarcomas, and testicular neoplasms being associated with pulmonary metastases, while head and neck SCC with primary lung cancer); nodule diameter and increase in size during follow-up [82, 87–89, 91, 92, 95–97, 100]. Specifically, in the studies developed in cohorts of patients with a specific ETN (Table 3), nodule size ≥ 10 mm [76, 93, 94, 96, 98] and disease stage [76, 81, 94–96, 98] have been most commonly addressed as predictors of malignancy.

Based on the observed results, different follow-up strategies have been suggested. Mano et al. indicated that the detection of IPNs ≤ 10 mm at preoperative CT scan should not delay curative treatment [93], while Xu et al. and Brookes MJ et al. emphasized the importance of close monitoring for patients with pulmonary nodules identified at the staging phase [73, 99]. However, these studies were retrospective in design and derived from single centre cohorts, thus limiting somewhat the strength of their recommendations [73, 93, 99]. Nuijens et al. developed and validated a clinico-radiological prediction model in patients affected by colorectal cancer with IPNs detected at baseline CT; based on four risk factors (location of primary tumour, pathological nodal stage, size of the largest nodule, and extrapulmonary synchronous metastases at diagnosis), a nomogram was constructed to express the percentage risk of IPNs being metastases (AUC of 0.79 in the validation dataset), which may prove useful in guiding the follow-up schedule [96].

To the best of our current knowledge, systematic reviews in this field have been developed in patients affected by resectable pancreatic adenocarcinoma [72], colorectal cancer [74, 77], head and neck SCC [100], and bone sarcoma [97]. Kuan et al., in their recent systematic review on resectable pancreatic adenocarcinoma, did not demonstrate potential associations between nodule features and probability of malignancy, while pointing out that the presence of pulmonary nodules on preoperative CT did not affect the overall survival (OS) after surgery; for these reasons, their detection should not prevent patients from undergoing curative surgery, although the importance of strict postoperative nodule surveillance is emphasized [72]. Data from the two recent systematic reviews regarding colorectal cancer [74, 77] demonstrated that the largest portion of IPNs turned out to be benign; coupled with the inability to rely on CT features alone for nodule characterization, this finding led Nordholm-Carstensen et al. to state that the detection of IPNs should not delay the intent of curative surgery of the primary tumour [74]. On the contrary, in patients affected by bone sarcoma, the likelihood of IPNs representing metastasis is non-negligible (45.0%), and close follow-up is advised for nodules > 5 mm [97].

Talwar et al. applied three validated models, namely the Mayo Clinic [101], the Veteran Association (VA) [102], and the McWilliams [103], in order to assess the probability of malignancy of small pulmonary nodules (≤ 12 mm in diameter) in a non-screening population that included patients with previous history of malignancy. However, the application in such a cohort resulted in a significantly lower performance of all the three models compared to the studies from which they originated (AUC Mayo: 0.58 vs 0.8; VA: 0.62 vs 0.79; McWilliams: 0.82 vs 0.94) [85, 104], therefore discouraging their application in clinical practice in this patient category. In the study by Soardi et al., the Bayesian Inference Malignancy Calculator (BIMC) model, when applied to the general population including patients with previous history of malignancy, showed a higher accuracy in risk stratification of solitary pulmonary nodules compared to the Mayo Clinic model (AUC 0.880 vs 0.604; *p* < 0.0001), potentially favouring a wider application of this model also in patients with extra-thoracic cancer history [85, 105].

**Fig. 11:**
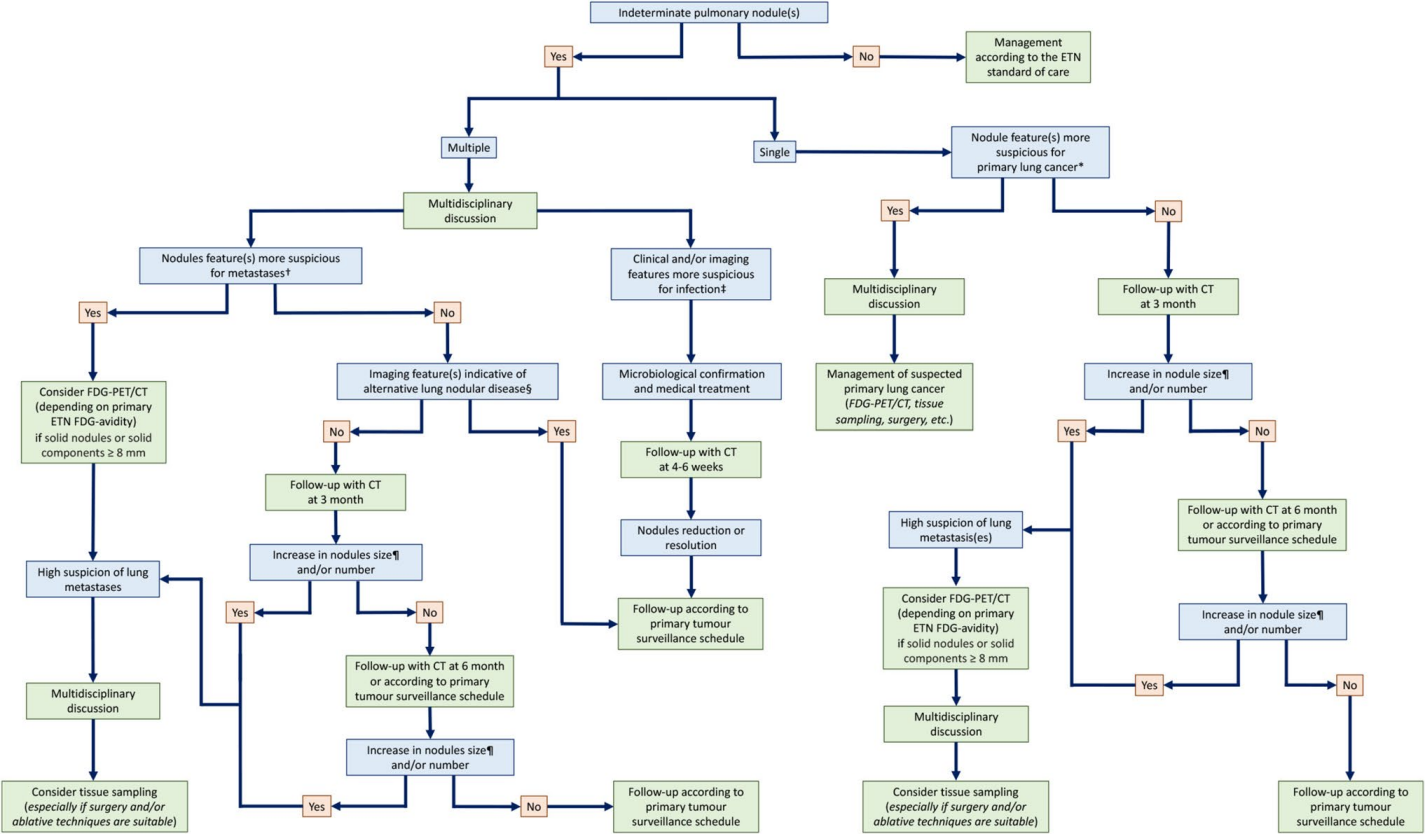
Proposal of diagnostic flowchart for IPN(s) management in patients with ETNs. Proposed diagnostic flowchart for indeterminate pulmonary nodule(s) (IPNs) in patients affected by extra-thoracic neoplasms (ETNs). This diagnostic algorithm should be regarded as a guide only. A tailored approach in a multidisciplinary context should be ensured, taking into account the risk factors related to both primary tumour (e.g., histology, stage) and patient (e.g., laboratory testing, symptoms, oncologic treatment). *See Table 1. †See text (Sect. “Introduction”) and Table 1. ‡Fever, productive cough, leucocytosis, C-reactive protein elevation, subsolid density, multiplicity and clustering, bronchial wall thickening/mucoid impaction, centrilobular nodularity, COPD (among the others). §OP, sarcoidosis, DIPNECH (among the others). ¶Increase in average diameter by at least 2 mm. ETN, Extra-thoracic neoplasm; CT, Computed Tomography; FDG-PET/CT, Fluorodeoxyglucose Positron Emission Tomography/Computed Tomography

Due to the abovementioned data, we favour a *case-by-case* approach for the management and follow-up of IPNs in patient with known ETNs [3]. The diagnostic algorithm should be tailored based on risk factors [76, 93] related to the primary tumour (location, histotypes and stage) and patient himself (e.g., presence of respiratory symptoms, laboratory testing, types and time elapsed from oncologic treatment), in the context of a multidisciplinary approach in which radiologists with experience in thoracic and oncological imaging should play a leading role. CT nodule features should be used as a guide for selecting the best further diagnostic option for each patient, with either non-invasive (such as PET/CT) or invasive (biopsy or surgical resection) approach. As a general rule, with respect to the general population, the follow-up interval should be shortened and invasive procedure promoted, especially if metastases can be treated with surgery or ablative techniques [72, 73, 76, 88, 91, 93]. Taking into consideration the high incidence of infective/inflammatory nodules occurring in cancer patients ongoing chemo- and/or radiation therapy, when clinical and imaging suspicious (e.g., new-onset subsolid nodule) of infection is raised [98], a chest CT follow-up at 4–6 weeks is advised [3]. In case of indeterminate nodule(s) detected on the staging CT, a follow-up after 3 months could be considered as appropriate, with a subsequent 6-month evaluation, if not indicated otherwise according to the primary tumour surveillance schedule [3, 81]. Of course, an increase in size (increase in mean diameter by at least 2 mm) [106] and/or in number during follow-up can be considered related to the metastatic nature of nodule(s) [77, 81], while in case of unchanged findings a total follow-up of at least 2 years [75] or equal to the general population can be considered appropriate, according to the guidelines for IPN(s) in the general population [41]. Figure 11 shows the proposed diagnostic flowchart for IPNs in patients with known ETNs.

## Conclusions

Radiologists must be aware of the variable imaging appearance of pulmonary metastases and the possible differential diagnoses in patients with ETNs, taking into account the clinical data and working synergistically with clinicians, oncologists, radiation oncologists, and surgeons, as part of a multidisciplinary cancer team. In this context, characterization and management of IPNs can be particularly challenging for radiologists, owing the lack of guidelines and validated prediction models. In this increasingly urgent task, the application of novel imaging modalities and approaches to imaging analysis (radiomics and AI) on large cancer populations might help in the definition of predictive models suitable to be integrated into clinical practice in the near future.

## Data Availability

Not applicable.

## Abbreviations

CT: Computed Tomography
ETN(s): Extra-thoracic neoplasm(s)
CXR: Chest X-ray
OP: Organizing pneumonia
DIPNECH: Diffuse idiopathic pulmonary neuroendocrine cell hyperplasia
MIP: Maximum intensity projection
MinIP: Minimum intensity projection
SPN(s): Solitary pulmonary nodule(s)
SCC: Squamous cell carcinoma
PLCH: Pulmonary Langerhans cell histiocytosis
PH: Pulmonary hamartoma
RCC: Renal cell carcinoma
AI: Artificial intelligence
NSCLC: Non-small cell lung cancer
PET: Positron Emission Tomography
AUC: Area under the curve
18F-FDG PET/CT: 18-Fluorine-fluorodeoxyglucose Positron Emission Tomography/Computed Tomography
IPN(s): Indeterminate pulmonary nodule(s)
BTS: British Thoracic Society
STR: Society of Thoracic Radiology
PLC(s): Primary lung cancer(s)
Yr(s): Year(s)
OR: Odds ratio
Pts: Patients
PET/CT: Positron Emission Tomography/Computed Tomography
DFS: Disease-free survival
OS: Overall survival
CEA: Carcinoembryonic antigen
PPV: Positive predictive value
PFS: Progression-free survival
VA: Veteran Association
BIMC: Bayesian inference malignancy calculator
